# Correlation Between Body Mass Index (BMI) and Severity of Bronchial Asthma in Children: A Cross-Sectional Study

**DOI:** 10.7759/cureus.111536

**Published:** 2026-06-26

**Authors:** Palak Satija

**Affiliations:** 1 Paediatrics, Christian Medical College, Ludhiana, IND

**Keywords:** asthma severity, body mass index (bmi), bronchial asthma, global initiative for asthma (gina) guidelines, obesity

## Abstract

Background: Nutritional status is increasingly recognised as a key modifier of asthma severity in children. Both obesity and undernutrition may influence airway inflammation and disease course through distinct mechanisms.

Objective: To assess the nutritional status of children with bronchial asthma and to correlate it with the severity of asthma, with subgroup analysis by sex.

Methods: A cross-sectional study with prospective data collection enrolled 42 children aged 5-18 years with physician-diagnosed bronchial asthma at a tertiary paediatric centre (June 2020 to December 2021). Asthma severity was graded using the Global Initiative for Asthma (GINA) criteria. Nutritional status was assessed by body mass index (BMI) for age plotted on the World Health Organization (WHO) gender-specific growth charts. Associations were tested with Fisher's exact test; a two-sided p-value of less than 0.05 was considered significant.

Results: Of the 42 children, 25 (59.52%) had a normal BMI, 8 (19.05%) were overweight or obese, 7 (16.67%) were underweight, and 2 (4.76%) were at risk of overweight. Increasing BMI was significantly associated with greater asthma severity (p=0.007). Overweight or obese children predominantly had moderate persistent asthma (four of eight; 50.00%). The association was statistically significant in boys (p=0.012), but not in girls (p=0.30).

Conclusion: Higher BMI significantly correlates with greater asthma severity in this cohort of school-age children, driven by a more pronounced effect in boys. Given the exploratory nature of this study, these findings are hypothesis-generating. While routine nutritional assessment is a reasonable component of paediatric asthma care, prospective studies are required to demonstrate whether weight optimization directly improves asthma outcomes.

## Introduction

Bronchial asthma is the most common chronic respiratory disease in children, affecting an estimated 262 million people globally and approximately 6% of Indian children [1]. It is characterised by chronic airway inflammation, hyper-responsiveness, and variable airflow obstruction. While allergen sensitisation and viral infections are classical triggers, nutritional status has gained increasing recognition as an important and modifiable determinant of asthma severity [2].

A positive association between elevated body mass index (BMI) and asthma prevalence is well documented. Obesity promotes a systemic pro-inflammatory state through upregulation of adipokines and pro-inflammatory cytokines, including leptin, interleukin-6 (IL-6), and tumour necrosis factor-alpha (TNF-α), that augment airway inflammation independent of atopic mechanisms [3]. This interaction underlines the clinical emergence of a distinct "obese asthma phenotype" - characterized by unique mechanical and inflammatory changes, more frequent and severe asthma episodes, and a poorer response to standard controller therapies. Consequently, identifying nutritional modifiers early is essential to understanding variations in disease presentation [4].

Conversely, undernutrition in asthmatic children, mediated through chronic systemic inflammation and reduced exercise tolerance, may impair lung development and weaken immune defence mechanisms. Despite this bidirectional relationship, data specifically correlating BMI category with asthma severity in Indian children remain limited.

This exploratory study was conducted to assess the nutritional status of children with physician-diagnosed bronchial asthma and to establish its correlation with asthma severity, with a subgroup analysis by sex.

## Materials and methods

Study design and setting

A cross-sectional study with prospective enrolment and data collection was conducted at the Department of Paediatrics, Christian Medical College and Hospital (CMCH), Ludhiana, Punjab, India, from June 2020 to December 2021. The design was cross-sectional in that nutritional status and asthma severity were each assessed at a single point per participant. No longitudinal follow-up was performed. Ethical approval was obtained from the Institutional Ethics Committee; written informed consent was obtained from parents, and assent was obtained from children aged 12 years and older.

Participants and sample size

Children aged 5-18 years with physician-diagnosed bronchial asthma were enrolled consecutively after screening for eligibility. Children with chromosomal or genetic syndromes affecting growth, chronic debilitating illness, neurological disability, or structural airway diagnoses (cystic fibrosis, primary ciliary dyskinesia) were excluded. No formal a priori sample size calculation was performed; all eligible children presenting during the defined study period were enrolled as a convenience sample. The resulting sample of 42 children is acknowledged as a limitation of the study, rendering this work strictly exploratory.

Assessment and confirmation of asthma diagnosis and severity

The diagnosis of bronchial asthma was established by the treating paediatrician on the basis of a compatible clinical history (recurrent wheeze, cough, breathlessness, and chest tightness), physical examination, and documented response to asthma therapy, in accordance with the Global Initiative for Asthma (GINA) criteria. Asthma severity was classified by the treating physician according to the symptom-based criteria of the GINA 2021 report into four grades: intermittent, mild persistent, moderate persistent, and severe persistent [5]. Due to age-related compliance limits and resource constraints at the time of evaluation, spirometry was not uniformly utilized for all eligible children; hence, it did not systematically contribute to the diagnosis or severity classification.

Nutritional assessment

Height and weight were measured using standardised techniques. Standing height was measured to the nearest 0.1 cm using a calibrated stadiometer, and weight to the nearest 0.1 kg using a calibrated weighing scale, with the child in light clothing and without footwear. Each measurement was taken twice, and the mean value was recorded. BMI was calculated as weight in kilograms divided by height in metres squared (kg/m²) and plotted on WHO gender-specific BMI-for-age charts [6]. Children were classified as underweight (below the 5th percentile), normal (5th-84th percentile), at risk of overweight (85th-94th percentile), or overweight/obese (at or above the 95th percentile). Owing to the small number of children in the highest BMI categories, the overweight and obese groups were combined into a single "overweight/obese" category to preserve statistical power and ensure adequate expected cell counts for Fisher's exact test; this grouping is consistent with their shared adiposity-driven pathophysiology.

Statistical analysis

Categorical data are presented as numbers and percentages. Associations between nutritional status (BMI category) and asthma severity (GINA grade) were assessed using Fisher's exact test, chosen because of the small sample size and low expected cell counts. To enhance interpretability beyond p-values, effect sizes for these categorical associations were evaluated using Cramer's V. Subgroup analyses by sex were performed. Because of the limited sample size and sparse cells, multivariable modelling was not undertaken (see Limitations). Statistical Product and Service Solutions (SPSS, version 21.0; IBM SPSS Statistics for Windows, Armonk, NY) was used; a two-sided p-value of less than 0.05 was considered statistically significant.

## Results

Participant characteristics

Forty-two children aged 5-18 years were included (mean age: 12.1 ± 3.5 years). Boys predominated (27 of 42; 64.29%; male-to-female ratio: 1.9:1). By asthma severity, 16 children (38.10%) had intermittent asthma, 17 (40.48%) had mild persistent asthma, and nine (21.43%) had moderate persistent asthma; notably, no child in this study population had severe persistent asthma. Severity was higher in boys, although this difference did not reach statistical significance.

Nutritional status

The majority of children (25 of 42; 59.52%) had a normal BMI. Overweight or obesity was found in eight children (19.05%), seven (16.67%) were underweight, and two (4.76%) were at risk of overweight. Overweight/obesity was predominantly a male phenomenon (eight of 27 boys, 29.63%, versus none of the girls); girls with asthma had a better overall nutritional status than boys. The full BMI classification, overall and by sex, is shown in Table 1.

**Table 1:** BMI classification of asthmatic children aged five years and older (N=42). BMI-for-age was plotted on WHO gender-specific growth charts. BMI: body mass index; WHO: World Health Organization.

BMI Category	Total (N=42), n (%)	Male (n=27), n (%)	Female (n=15), n (%)
Underweight	7 (16.67%)	4 (14.81%)	3 (20.00%)
Normal	25 (59.52%)	15 (55.56%)	10 (66.67%)
At risk of being overweight	2 (4.76%)	0 (0%)	2 (13.33%)
Overweight/Obese	8 (19.05%)	8 (29.63%)	0 (0%)

Correlation of BMI with asthma severity: overall

A statistically significant positive correlation was observed between increasing BMI and asthma severity (p=0.007; Fisher's exact test), with a moderate-to-strong effect size demonstrated by a Cramer's V of 0.44 (Table 2). Children who were overweight or obese predominantly had moderate persistent asthma (four of eight; 50.00%), compared with only 20.00% of children with a normal BMI. In contrast, four of seven underweight children (57.14%) had intermittent asthma, the mildest category. No case of severe persistent asthma was recorded in any BMI group.

**Table 2:** Association of asthma severity (GINA grade) with BMI category - all children aged five years and older (N=42). p=0.007 (Fisher's exact test), statistically significant. GINA: Global Initiative for Asthma; OW: overweight; BMI: body mass index

GINA Grade	Underweight (n=7)	Normal (n=25)	At risk OW (n=2)	Overweight/Obese (n=8)	Total (N=42)
Intermittent	4 (57.14%)	9 (36.00%)	1 (50.00%)	2 (25.00%)	16 (38.10%)
Mild persistent	3 (42.86%)	11 (44.00%)	1 (50.00%)	2 (25.00%)	17 (40.48%)
Moderate persistent	0 (0%)	5 (20.00%)	0 (0%)	4 (50.00%)	9 (21.43%)
Severe persistent	0	0	0	0	0

Subgroup analysis by sex

Among boys aged five years and older (n=27), a statistically significant association between increasing BMI and greater asthma severity was demonstrated (p=0.012; Table 3). All overweight or obese boys had moderate persistent disease. Boys with underweight or normal BMI were mostly in the intermittent or mild persistent categories.

**Table 3:** Association of asthma severity with BMI - boys aged five years and older (n=27). p=0.012 (Fisher's exact test), statistically significant. GINA: Global Initiative for Asthma; OW: overweight; BMI: body mass index

GINA Grade	Underweight (n=4)	Normal (n=15)	Overweight/Obese (n=8)	Total (n=27)
Intermittent	2 (50.00%)	5 (33.33%)	2 (25.00%)	9 (33.33%)
Mild persistent	2 (50.00%)	8 (53.33%)	2 (25.00%)	12 (44.44%)
Moderate persistent	0 (0%)	2 (13.33%)	4 (50.00%)	6 (22.22%)

Among girls aged five years and older (n=15), the majority had intermittent-to-mild persistent asthma regardless of BMI category, and no girl was overweight or obese (Table 4). The association between BMI and asthma severity was not statistically significant in girls (p=0.30), suggesting that the BMI-severity effect was driven predominantly by the male subgroup.

**Table 4:** Association of asthma severity with BMI - girls aged five years and older (n=15). p=0.30 (Fisher's exact test), not statistically significant. No girl was in the overweight/obese category. GINA: Global Initiative for Asthma; OW: overweight; BMI: body mass index

GINA Grade	Underweight (n=3)	Normal (n=10)	At risk OW (n=2)	Total (n=15)
Intermittent	2 (66.67%)	4 (40.00%)	1 (50.00%)	7 (46.67%)
Mild persistent	1 (33.33%)	3 (30.00%)	1 (50.00%)	5 (33.33%)
Moderate persistent	0 (0%)	3 (30.00%)	0 (0%)	3 (20.00%)

## Discussion

This study demonstrates a significant positive correlation between increasing BMI and asthma severity in school-age children, with a sex-specific effect that was predominantly seen in boys. To our knowledge, few Indian studies have specifically examined this BMI-asthma severity relationship within a paediatric cohort stratified by sex.

The predominance of overweight/obesity among boys with asthma (29.63% versus 0% in girls) and the significant BMI-severity correlation in boys (p=0.012) likely reflect a combination of factors. First, physically active boys with worsening asthma may progressively reduce activity, leading to weight gain. Second, obesity itself intensifies airway inflammation through cytokine upregulation (leptin, IL-6, and TNF-α), creating a self-reinforcing cycle [7]. Third, the mechanical effects of abdominal adiposity reduce functional residual capacity (FRC) and worsen expiratory airflow limitation [8]. Obesity is increasingly recognised as a distinct asthma phenotype with its own inflammatory and mechanical underpinnings and a poorer response to standard controller therapy [9]. This interaction reinforces the concept of the obese asthma phenotype as a distinct clinical entity. The absence of a significant association in girls may reflect the lower burden of obesity in this cohort's female subgroup, which limited statistical power.

Crucially, because only eight children in our entire cohort were overweight or obese, these specific observations must be regarded as strictly hypothesis-generating. Furthermore, because the full asthma severity spectrum was not represented - no cases of severe persistent asthma were documented in our convenience sample - caution must be exercised when interpreting these correlations, as they cannot be generalized to patients with severe persistent disease.

Our findings align with those of Sharif-Askari et al., who reported moderate-to-severe asthma in 33% of overweight or obese children compared with 12% of normal-weight or underweight children [10]. Lang et al. demonstrated a statistically significant worsening of asthma severity with increasing BMI [11], and Somashekar et al. showed significant decrements in peak expiratory flow rate (PEFR) and forced expiratory volume in one second (FEV1) with rising BMI [12]. A meta-analysis by Chen et al. estimated a 20% increase in asthma incidence in overweight children, rising to an approximately twofold increase in obese children, with a stronger effect in boys [13]. Consistent with our observations, overweight and obese asthmatic children have also been reported to respond less well to inhaled corticosteroids [14], which may further contribute to the more severe phenotype.

Underweight children in our cohort predominantly had intermittent, milder disease. While chronic undernutrition can impair immune function and respiratory muscle strength, the milder asthma phenotype in underweight children may also reflect a selection bias toward less severe disease in this nutritional category, or a possible protective role of lower airway intraluminal pressure. This finding requires confirmation in larger cohorts.

Confounding factors

Several potentially important confounders were not measured or adjusted for and may have influenced the observed association. These include atopic status and allergic comorbidities (allergic rhinitis, eczema), the use of and adherence to inhaled corticosteroids and other controller therapy, the level of habitual physical activity and aerobic fitness, dietary pattern, socioeconomic status, exposure to environmental tobacco smoke and other indoor and outdoor pollutants, family history of asthma and atopy, and pubertal stage (which influences both adiposity and airway calibre and may underlie part of the observed sex difference). The relationship reported here should therefore be interpreted as an association rather than evidence of an independent or causal effect.

Limitations

This study has several limitations. It was a single-centre study with a small sample (N=42), and only eight children were overweight or obese, which limits statistical power, widens confidence intervals, and reduces generalisability. Because the study is explicitly exploratory, participants were enrolled as a convenience sample, and no a priori sample size calculation was performed. The cross-sectional design fundamentally precludes inferences about temporality or causation. Because of the small numbers and sparse cells, only univariate (bivariate) analyses were performed; multivariable logistic regression, which would have allowed assessment of the independent association between BMI and asthma severity after adjustment for confounders, was not feasible without a high risk of overfitting and unstable estimates. The female subgroup contained no overweight or obese children, so the sex-specific comparison should be interpreted cautiously. Finally, the absence of spirometric data and a lack of representation across the full asthma severity spectrum mean these exploratory results require confirmation in larger, multicentre, prospective studies using multivariable modeling.

Clinical implications

From a clinical standpoint, these exploratory data indicate that tracking BMI and providing basic nutritional guidance are reasonable components of paediatric asthma management. Overweight or obese children with asthma represent a practical target for lifestyle advice aimed at supporting general health and mitigating long-term cardiometabolic risk.

## Conclusions

In this cross-sectional study of school-age children with bronchial asthma, nutritional status as measured by BMI was significantly correlated with asthma severity within the sample population. Overweight and obese children, and boys in particular, experienced more severe disease, whereas underweight children predominantly had milder, intermittent asthma. These findings reinforce the concept of obesity as a clinically relevant modifier of the paediatric asthma phenotype and highlight a sex-specific pattern that warrants further study. While these findings are hypothesis-generating and support the clinical concept of the obese asthma phenotype, the cross-sectional design and unadjusted confounders preclude establishing any causal link. Integrating routine anthropometric assessment and basic weight management into comprehensive paediatric asthma care remains a reasonable practice. Adequately powered, multicentre, longitudinal studies employing multivariable analysis are required to confirm the independent contribution of adiposity to asthma severity and to evaluate whether weight optimisation improves asthma outcomes in this population.
